# Canine Vaginal Cytology: A Revised Definition of Exfoliated Vaginal Cells

**DOI:** 10.3389/fvets.2022.834031

**Published:** 2022-03-24

**Authors:** Felix Reckers, Robert Klopfleisch, Vitaly Belik, Sebastian Arlt

**Affiliations:** ^1^Clinic for Animal Reproduction, Freie Universitaet Berlin, Berlin, Germany; ^2^Veterinary Pathology, Freie Universitaet Berlin, Berlin, Germany; ^3^Veterinary Epidemiology and Biometry, Freie Universitaet Berlin, Berlin, Germany

**Keywords:** exfoliative vaginal cytology, vaginal smear, estrus cycle, vaginal cell tutorial, digital microscopy, female dog

## Abstract

Vaginal cytology is an important examination method in the context of gynecological disorders and cycle staging in the bitch. While collection and preparation of samples are easy, the evaluation appears to be challenging. Inconsistent definitions of cell attributes such as size, cornification and the appearance of the nucleus have been published. The aim of the project was to develop a tutorial for vaginal cell determination. To get a deeper insight into the use of cytology in practice, an online survey was distributed to veterinarians interested in small animal reproduction. Participants were asked to define eight cells and answer questions. The agreement of the 16 participants, working in eight different countries, determining the cells was poor (κ = 0.412). Eleven respondents stated that vaginal cytology has a low reliability. Nevertheless, 13 participants use this tool regularly. The tutorial was developed as a flowchart based on the survey results, scientific literature and own measurements. It guides the user systematically through the evaluation of specific cell characteristics. An evaluation of the results of five raters with difference experience levels led to a high agreement (κ = 0.858). Vaginal cytology is a useful diagnostic tool, but it seems helpful to standardize the determination of cell types.

## Introduction

Gynecological examinations belong to the standard procedures in veterinary medicine. The findings are important in the context of breeding management (1). Finding the day of ovulation during estrus is essential for an appropriate timing of mating or insemination and, thus, a high fertility (2). Symptoms such as acceptance of a male for mating (3), the sanguineous vulvar discharge (3), and the edema of the vulva (4) are signs of proestrus and estrus. The symptoms which can be observed via vaginoscopy are color, the intensity of edema and moisture of the vaginal mucous membrane (4, 5). Since some bitches do not show overt behavioral signs (6) it can be helpful to determine other stages of the estrus cycle, i.e. proestrus or diestrus. Also abnormal estrus cycle patterns, such as early or late ovulation, silent heats or split heats (7) can be diagnosed. External symptoms of proestrus are turgid vulvar swelling and sanguineous vulvar discharge (8). The discharge contains a large number of erythrocytes due to diapedesis through the uterine capillaries due to estrogen effect (9). The erythrocytes can also be found in the vaginal smear (10). The diestrus is characterized by slight mucoid discharge which often contains a large number of neutrophils in early stage. The bitch will not allow mounting or breeding of male dogs anymore (8). Furthermore, vaginal cytology belongs to the standard gynecological examinations because it enables insights into potential estrogen influences and the health status of the vagina (11). Sampling of cells, preparing and staining of a smear for vaginal cytology requires only few skills (12) and can be performed rapidly and inexpensively in daily practice (13, 14). However, the analysis and interpretation of the smears may be affected by several factors. Different sampling and staining procedures may bias evaluation results (15). In addition, the skills of the observer, transition between the different cell stages as well as individual characteristics of the bitch, such as a massive occurrence of erythrocytes, may impede the evaluation of vaginal smears (5).

To the best of our knowledge, no uniform standardized definition of canine vaginal cell types has been published, yet. A closer look into the literature reveals that authors suggest different parameters and definitions of the type of cells, for example regarding the diameter of the different cell types (Table 1).

**Table 1:** The table shows the diameter of the different cell types according to several authors.

**Type of cell**					
**Author**	**Basal cell**	**Parabasal cell**	**Intermediate cell**	**Superficial cell**	**Squamous cell**
Johnston et al. (16)	Usually not exfoliated	10–20 μm	20–30 μm	30–75 μm	-
Dreier (17)	10–20 μm	15–25 μm	20–30 μm	35–60 μm	Same size as superficial cells
Wehrend (5)	10–20 μm, usually not exfoliated	10–20 μm	20–50 μm	No diameter measurable because of folds	-
Bostedt et al. (18)	10–15 μm	15–30 μm	25–30 μm	40–60 μm	Same size as superficial cells
Antonov (11)	10–20 μm, usually not exfoliated	15–25 μm	20–30 μm	30–75 μm	-

Besides the diameter, the presence and extent of cornification are important aspects for the determination of vaginal cells. The cornification refers to the degenerative process of cells in higher layers in stratified squamous epithelia (16). Different authors give different definitions of cell types based on the extent of cornification (Table 2). A majority of the authors agree that basal, parabasal and intermediate cells are not cornified at all (16, 20).

**Table 2:** Presence and extent of cornification of different types of vaginal cells according to different authors.

		**Definition of the presence and extent of cornification**			
**Author**	**Basal cell**	**Parabasal cell**	**Intermediate cell**	**Superficial cell**	**Squamous cell**
Schutte (19)	-	Not cornified	Small intermediate cells: none Large intermediate cells: Not necessarily found	“not necessarily found”	“With a few exceptions always keratinized”
Johnston et al. (16)	Not cornified	Not cornified	Not cornified	Cornified	Cornified
Perez et al. (12)	-	Not cornified	Not cornified	“Partially cornified”	“Fully cornified”
Root Kustritz (20)	-	Not cornified	Not cornified	Cornified	Cornified
Wehrend (5)	Not cornified	Not cornified	Not cornified	“Increasing cornification”	Not specified
Bostedt et al. (18)	Not cornified	Not cornified	Not cornified	“Are subject to cornification”	“final stage of cornification”
Antonov (11)	-	-	-	-	Cornified

*“Not cornified” means that this cell type does not show signs of cornification. “-” means that the author did not specify the appearance of cornification of this cell type. Descriptions in hyphens are quotes from the respective author*.

Basal, parabasal and intermediate cells have a round unaltered nucleus (11, 19, 21). According to Johnston et al. (16) the area of the nucleus of an intermediate cell is >90 μm^2^. Other authors specify the diameter for the nucleus of an intermediate cell to be 7–11 μm (22), what corresponds to an area between approximately 38.5 μm^2^ and 95.0 μm^2^. Most authors agree that the nucleus is large and clearly discernible (19), prominent and appears normal (16). Superficial cells are described unanimously as cells having a pyknotic nucleus (12, 22, 23), which becomes eventually karyorrhectic (18) or karyolitic (17).

For most authors, the shape of the vaginal cells is an important aspect (22, 24, 25). However, other authors state that intermediate and superficial cells can be confused if they are defined by their shape, only (5, 16). The definition of squamous cells is also not explicitly clear. While some authors state that the nucleus is not visible (19, 20, 24), others observed that these cells are anucleated but often remainings of the disintegrated nucleus are still visible (18). Based on these heterogeneous definitions, it is likely that different evaluators come to different conclusions when interpreting vaginal smears (1). This has also been shown by Arlt (15).

An often-used method for the determination of the cycle stage is the determination of specific percentages of cell types. Some authors claim that a typical vaginal smear in estrus has 100% superficial cells and >80 % cells with pyknotic or absent nuclei (11, 26). Another definition of estrus is the presence of more than 90% superficial keratinized epithelial cells (13). Others define the cytological estrus by 100 % cornification with more than 50% anuclear squames (20). For the determination of diestrus based on specific percentages of exfoliated vaginal cells, the definitions by different authors show a high agreement. The onset of diestrus occurs when the number of superficial and squamous cells has decreased by at least 20% (10, 11, 13) and when a higher number of neutrophil granulocytes are present (23, 25). The heterogenous definitions of cell types and percentages of cells characteristic for specific cycle stages by different authors may impair cycle stage diagnoses and lead to a suboptimal agreement between different evaluators. Aim of this study was to learn more about how small animal reproduction experts define vaginal cells. In a second step, we wanted to develop more robust definitions of vaginal cells and evaluate a cell determination tutorial.

## Materials and Methods

### Preparation and Digitalization of Vaginal Cell Slides

In total, 52 vaginal smears were taken from 39 bitches for which health of the genital tract was confirmed. All bitches were presented for gynecological examination in the context of ovulation timing or routine gynecological health check. Bitches were aged between 1 and 14 years (median 5 years, interquartile range 3 and 6 years) and belonged to the breeds Siberian Husky, Continental Bulldog, Great Dane, Rottweiler, Golden Retriever, Afghan Hound, Dobermann (*n* = 2), Miniature Schnauzer (*n* = 3), Pomsky, Manchester Terrier (=2), French Bulldog (*n* = 2), Dachshund, Smooth Collie, Cross Breed (=5), Swiss Cattle Dog, Miniature Bullterrier, Pug (*n* = 2), Labrador (*n* = 3), Cairn Terrier, Kangal Shepherd Dog, Bernese Mountain Dog, Border Collie, Shiba Inu, German Shepherd, Leonberger, Monkey Terrier, and Berger Picard. The weight of the dogs ranged between 5 to 56 kg (median 20 kg, interquartile range 10 and 30.25 years). All smears used for this project were leftovers from clinical examinations.

All specimens were taken via an inserted sterile speculum (Proctovision, Karl Storz SE & CO. KG, Tuttlingen, Germany) as described by (11). The samples were collected by using a saline moistened, sterile cotton swab (12). The cotton swab (Medical applicator, Heinz Herenz Medizinbedarf GmbH, Hamburg, Germany) was introduced through the speculum (5) to collect cells from the caudodorsal surface of the vagina (23). After swabbing by gently rolling the dorsal vaginal wall, the swab was removed and rolled onto a glass slide (Objektträger ELKA, Glaswarenfabrik Karl Hecht GmbH & Co KG, Sondheim, Germany) (14). Routine Diff-Quick staining (Haema-Schnellfärbung, LT- Sys Eberhard Lehmann GmbH, Berlin, Germany) was performed after air drying (14). A coverslip (Deckgläser 32^*^22 mm, Glaswarenfabrik Karl Hecht GmbH & Co KG, Sondheim, Germany) was permanently fixed (Roti Histokitt II, Carl Roth GmbH & Co. KG, Karlsruhe, Germany) to the specimen (16). Bitches in different stages of the estrus cycle were chosen, so that all types of vaginal cells were represented.

For digitization the stained slides were scanned with Aperio CS2 (Leica Mikrosysteme Vertrieb GmbH Mikroskopie und Histologie, Wetzlar, Germany). The data were converted into Aperio Scan Scope Virtual Slide (svs) files and analyzed using the software program QuPath^®^. QuPath^®^ is an open-source software platform for whole slide image analysis (27). The program is able to show the slides in more than 400 × magnification and allows measurements of the area and diameter of nuclei or whole cells. It can also be used to label specific cells.

### Structure of the Survey

To reach veterinarians who are specialized in small animal reproduction the international Email list “Café Reprod” with around 150 members (personal information from the list administrator, spring 2021) worldwide was used for the distribution of the survey. No reminder was sent. The survey was open for 3 weeks.

**Figure 1:**
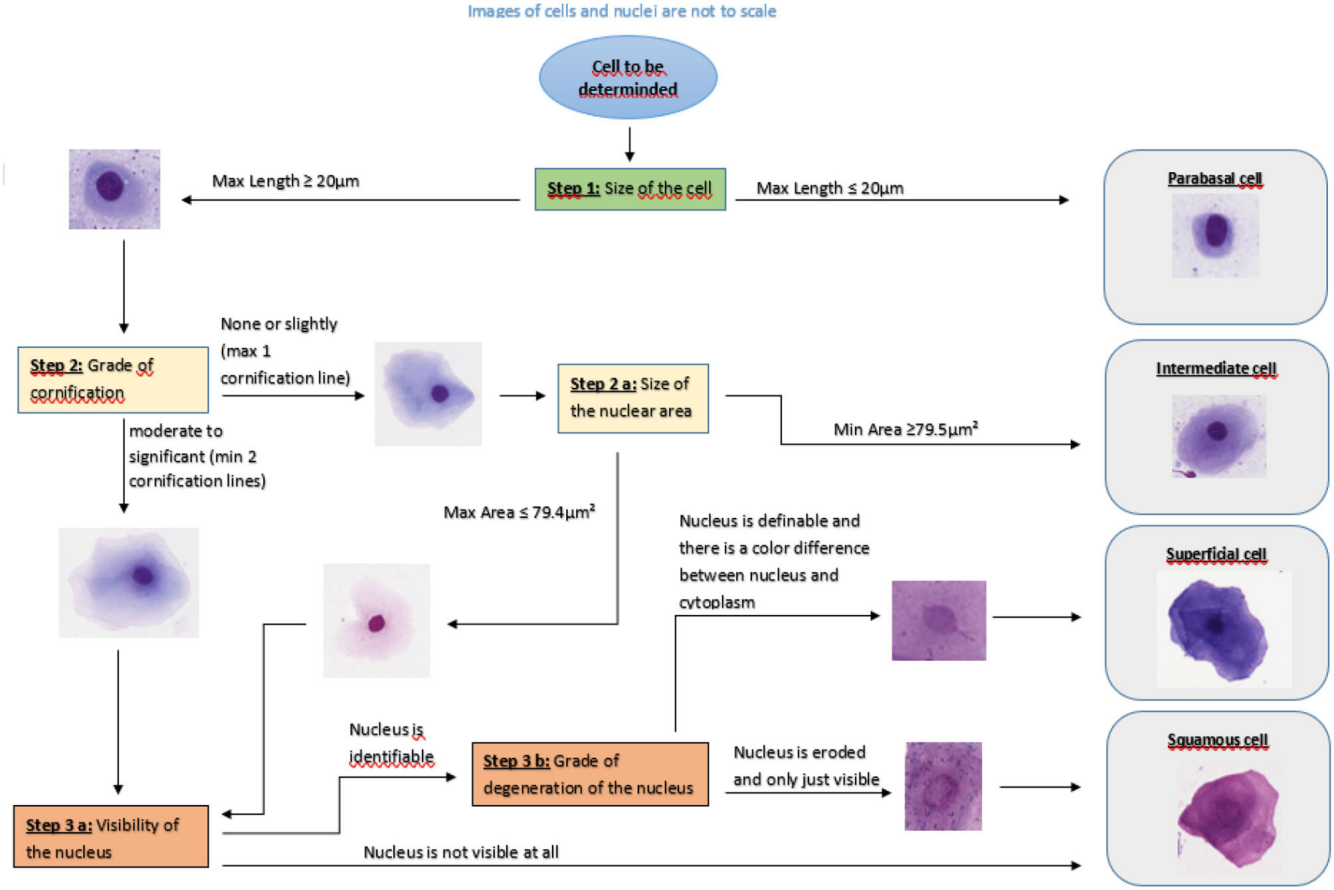
Flowchart for the determination of the cell type of exfoliated canine vaginal cells.

The survey consisted of three parts: In the first part, eight pictures of different vaginal cells were illustrated. Participants were asked to define cells as basal cell, parabasal cell, intermediate cell, superficial cell and squamous cell by ticking a box (see Figure 1).

In the second part, the participants were asked to answer the following questions by typing a short free text: How do you differentiate a parabasal cell from an intermediate cell? How do you differentiate an intermediate cell from a superficial cell? How do you differentiate a superficial cell from a squame?

Finally, questions about the practical work with vaginal cytology and about some personal information were asked.

### Development and Validation of a Tutorial for Vaginal Cell Determination

The information from the survey results and from scientific literature were analyzed. Based on this information and the digitized smears, definitions including specific parameters such as cell size and shape as well as nucleus size and shape were revised and evaluated for all vaginal epithelial cell types.

The tutorial was designed as a flowchart which aims to support determination of canine vaginal cells. For a first validation, five vaginal smears were used. The slides were chosen by the authors in order to ensure that all epithelial cell types were present.

On each slide, the first author assigned the numbers one to 100 to 100 cells by using QuPath^®^. Inclusion criteria for the selection of the cells were a clear and well visible structure of the cell. Cells which overlapped with other cells or structures were excluded. With the help of a random number generator (https://www.zufallsgenerator.net) 20 cells per slide were selected for the validation. This resulted in 100 cells in total.

All 100 cells were evaluated independently by five persons using the same computer and the same supporting material. No further information e.g., about the dog or stage of sexual cycle were given. The respondents were selected in order to represent raters with different levels of experience. Therefore, the raters were two students of veterinary medicine in their 5th year, two veterinarians working in the field of small animal reproduction for 12 and 24 months, respectively, and one diplomate (ECAR) for small animal reproduction. The first author supervised the process, did not take part in the evaluation and did not influence the evaluation. All evaluators received a list with the numbers of the cells to determine, a colored Din A4 version of the tutorial (Figure 1) and a form with a table in which they were asked to document the cell type according to the slide number and cell number.

### Statistical Analysis

Fleiss' Kappa was used, which measures the inter-rater reliability between more than two raters. For the calculation of Fleiss' Kappa in this project, R programming language was used (https://www.r-project.org/).

If *n*_*ij*_ is the number of raters who assigned the *i*-th subject (*i* = 1, …, *N*) to the *j*-th category (*j* = 1, …, *k*), then Fleiss' kappa is defined as


$$\kappa \;=\;\frac{\bar{P}-\overset{\bar{P}}{e}}{1-\overset{\bar{P}}{e}}$$

$$\begin{array}{l} Where\;\bar{P}=\frac{1}{N}\sum_{i=1}^{N}P_{i},\;P_{i}=\frac{1}{n\left(n-1\right)}\sum_{j=1}^{k}n_{ij}\left(n_{ij}-1\right),\\ \;{\bar{P}}_{e}=\sum_{j=1}^{k}p_{j}^{2},\;p_{j}=\frac{1}{Nn}\sum_{i=\;1}^{N}n_{ij}. \end{array}$$

## Results

### The Results of the Survey

**Figure 2:**
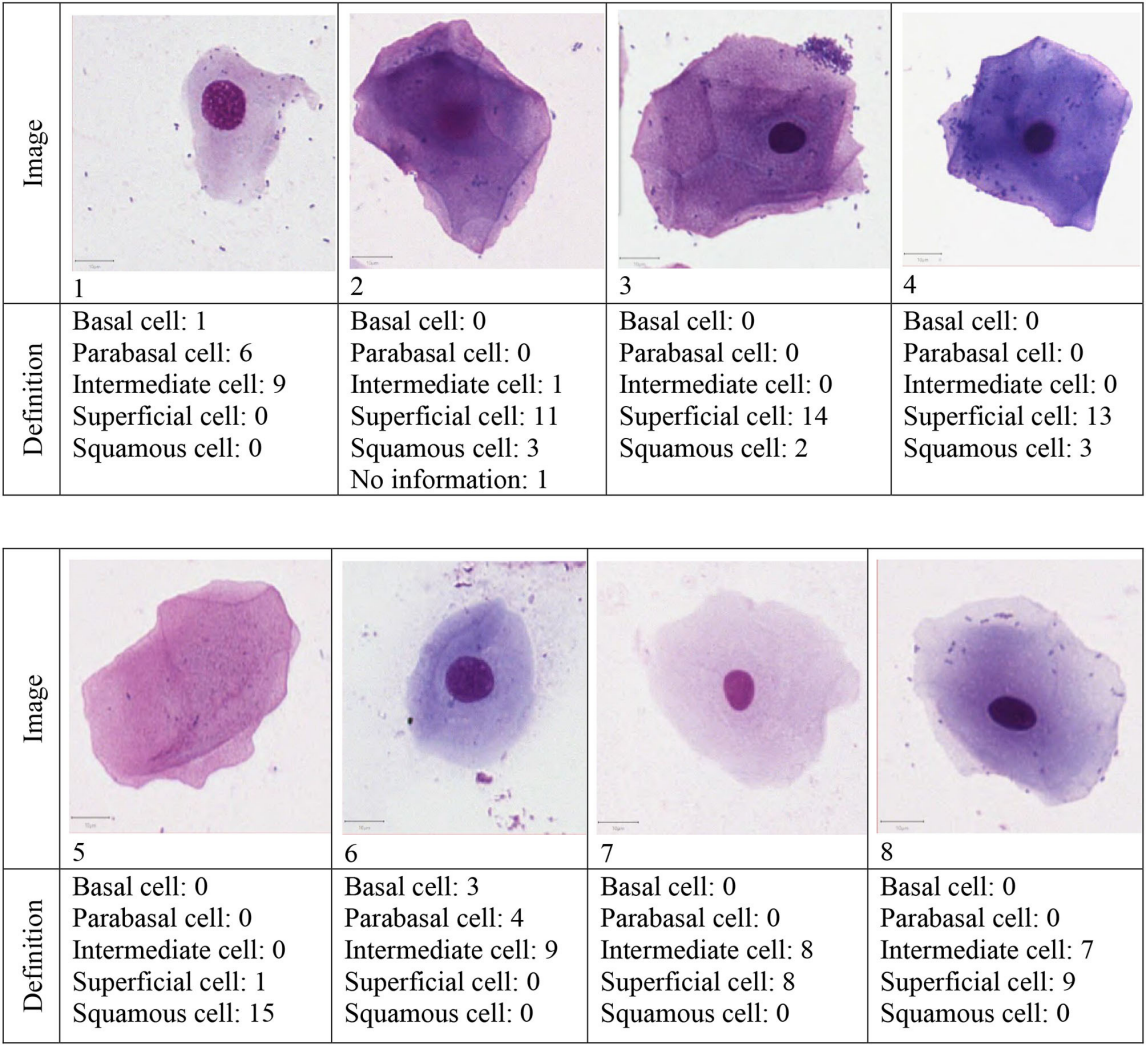
Results of the determination of eight canine vaginal cells by 16 veterinarians.

In total, 16 respondents completed and returned the survey. The definition of the eight presented cells by the respondents are presented in Figure 2.

Cell number one was defined as intermediate cell by nine respondents or as parabasal (*n* = 6) or basal cell (*n* = 1). Cells number two, three and four were identified by the most respondents as superficial cells. Cell number five had the highest accordance: all but one respondent defined it as a squamous cell. The definition of cell number six gave a more ambiguous result: nine respondents defined it as intermediate cell but seven were of the opinion that it is a parabasal or basal cell. Both, cells number seven and eight, were defined as intermediate cell by around half of the participants and as superficial cell by the other half. The calculated Fleiss' Kappa for the accordance of all 16 raters was κ= 0.412.

In the second part, the respondents were asked how they discriminate between cell types. For each of the three scenarios the participants named at least two parameters.

The first question was how they differentiate a parabasal cell from an intermediate cell. The most named parameters were size (*n* = 10) and shape (*n* = 10) of the cells. Nine respondents mentioned the ratio between the nucleus and the cytoplasm. The appearance of the nucleus (size and shape) was named by seven and cornification by one respondent.

The second question was how to differentiate an intermediate cell from a superficial cell. Shape (*n* = 11) and appearance of the nucleus (*n* = 11) were the main parameters for differentiation of the cells, followed by the size of the cell (*n* = 7). Parameters of minor importance seem to be the cytoplasm: nucleus ratio (*n* = 5), the cornification (*n* = 3) and the color of the cell (*n* = 2).

Finally, the participants were asked how they differentiate a superficial cell from a squamous cell. Mostly named parameters were the presence and appearance of the nucleus (*n* = 11). The shape (*n* = 3), the grade of cornification (*n* = 2) and the size (*n* = 1) had only a minor influence for the differentiation of superficial and squamous cells. Some of the participants stated there was no difference between the two cell types (*n* = 3).

The third part of the survey referred to the practical work and experience of the participants. The number of bitches the participants stated to see in the context of ovulation timing per year is median 125 (interquartile range 50–300).

The majority of the respondents indicated that they perform vaginal cytology in the context of ovulation timing (*n* = 13). Several respondents stated, however, that vaginal cytology is “Not reliable at all” (*n* = 5) or “Not reliable” (*n* = 6) in the context of ovulation timing. The minority chose the answer “Moderate” (*n* = 3) or the answer “Reliable” (*n* = 2). All participants agreed that progesterone measurement for ovulation timing is “Very reliable” (*n* = 8) or “Reliable” (*n* = 8). The respondents use immuno assays for progesterone measurement: Immulite^®^ (*n* = 8), Minividas^®^ (*n* = 3), TOSOH^®^ (*n* = 1), Hormonost^®^ ELISA kit (*n* = 3). In one case no answer was given.

Finally, the respondents were asked about their experience and person. The participants have worked as veterinarian for 24.1 (±10.2) years in mean. One respondent did not specify the years in practice but wrote that she or he has practiced “too long”. Asked for the highest degree they have achieved, the participants chose “Veterinarian” (*n* = 7), “Diplomate” (*n* = 6), “PhD” (*n* = 2) or “Specialist of reproduction” (*n* = 1). They work in the USA (*n* = 7), Sweden (*n* = 2), Germany (*n* = 1), Hungary (*n* = 1), Thailand (*n* = 1), Belgium (*n* = 1), Portugal (*n* = 1) and Australia (*n* = 1). One participant stated to work “worldwide”.

### The Structure of the Tutorial

The tutorial (Figure 1) was developed as a flowchart which enables following flowlines and assessing cell aspects step by step. The aim was to guide the user through the evaluation of relevant cell parameters. To support the decision process, sample images were included. These images were derived from the digitalized slides. The tutorial for the evaluation of a vaginal cell starts with the determination of the cell diameter. According to several authors, the maximum diameter of a parabasal cell is 20 μm. To validate this definition, vaginal smears from 10 bitches in anestrus or early proestrus were used. On each specimen 20 cells with a diameter smaller than 20 μm were measured and analyzed with QuPath^®^. This resulted in 200 cells in total. Not one cell showed cornification or changes in the appearance of the nucleus. Hence, the given definition is suitable and was, therefore, included into the tutorial.

**Figure 3:**
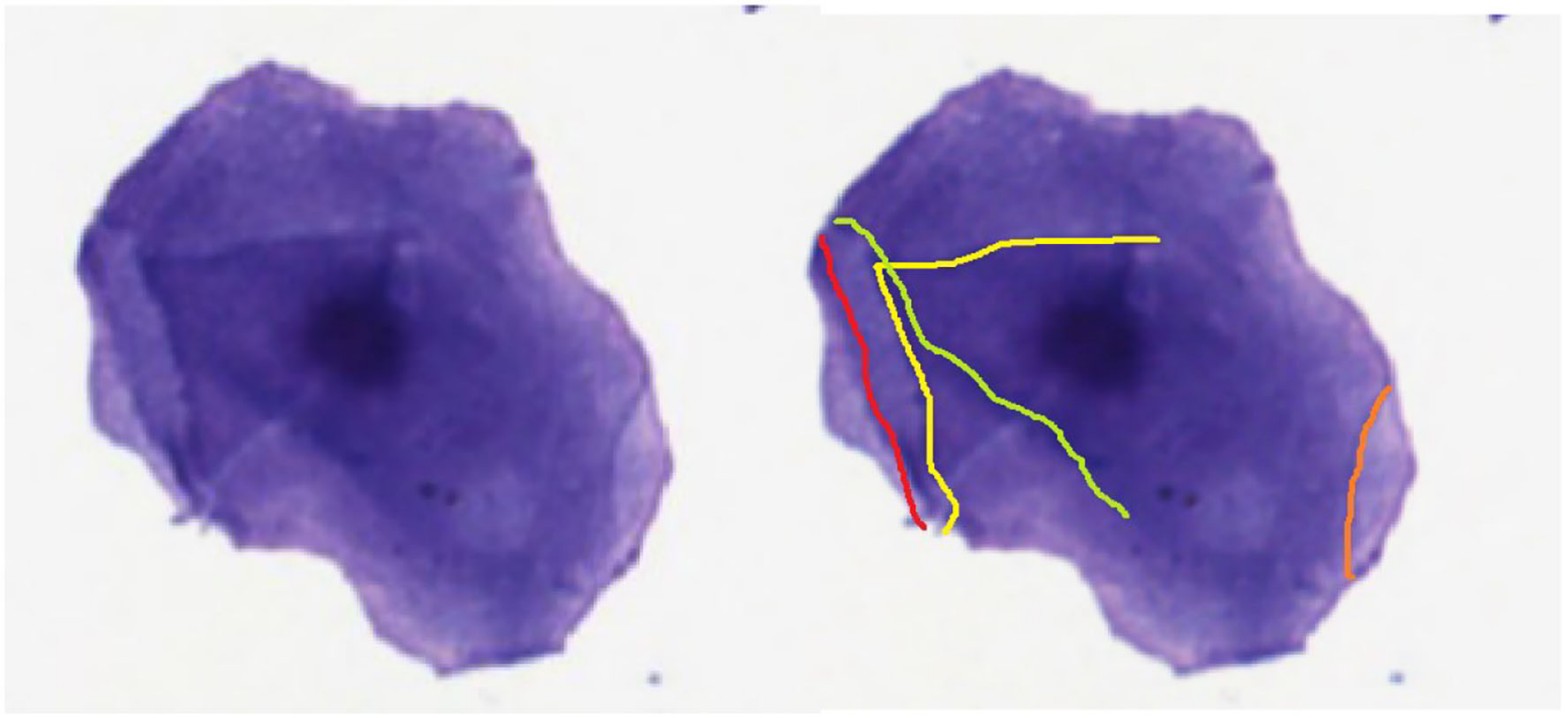
Cornification lines of a vaginal epithelial cell.

If the cell diameter exceeds 20.0 μm the user follows the flowline to the decision box “Step 2: Grade of cornification”. At step 2, the user can decide between the flowlines “none or slightly” and “moderate to significant”. These flowlines lead to decision box “Step 2.a.: Size of the nuclear area” or decision box “Step 3.a.: Visibility of the nucleus”, respectively. To standardize this decision about the grade of cornification, all cells with no or only one cornification line should be determined as cells with no or slight cornification (Figure 3).

**Figure 4:**
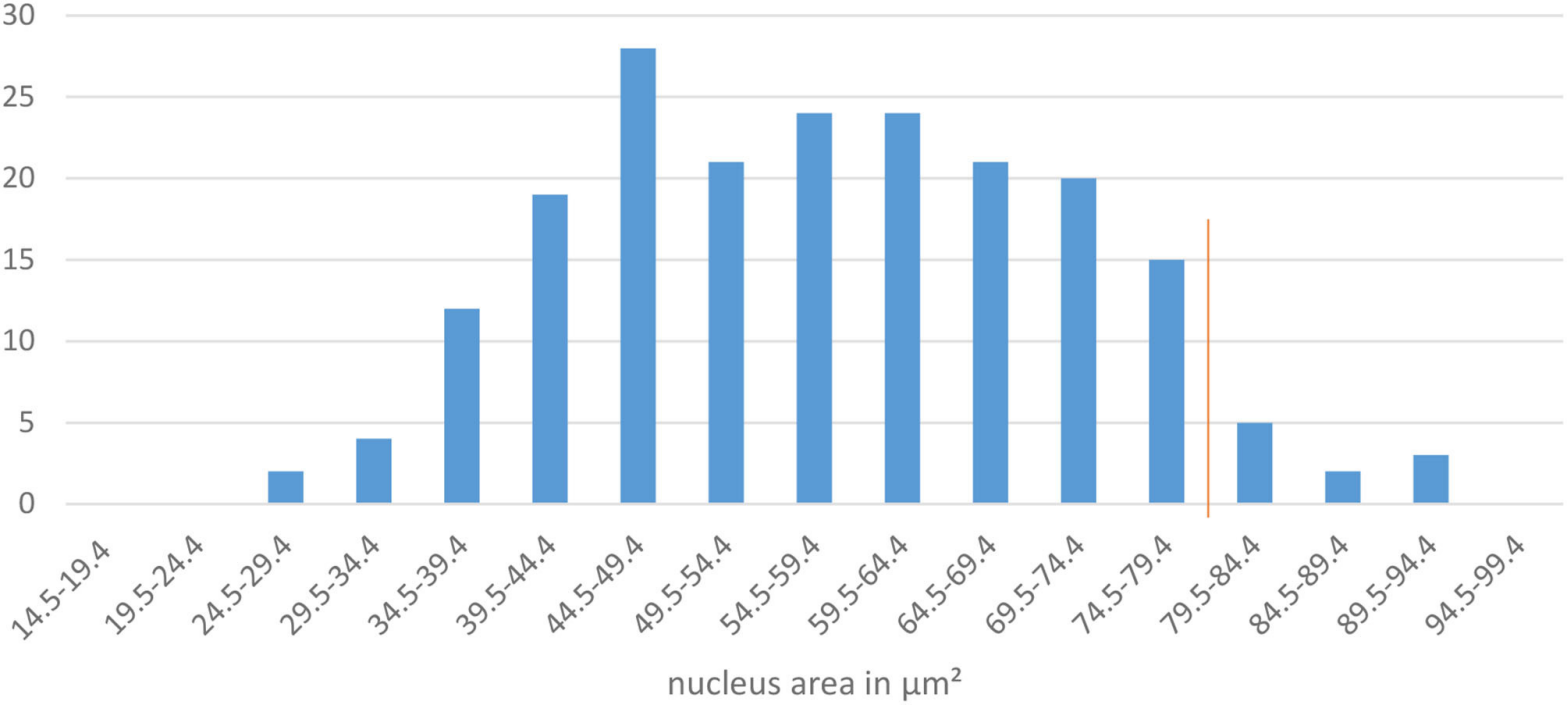
The diagram shows the area of the measured nuclei of cornified vaginal cells in μm^2^ and the frequency of measurements. The red line marks the 95 percentile.

The decision box 2.a. refers to the size of the nucleus. According to the above-named authors the area of the nucleus of an intermediate cell is large, round and unaltered. The nucleus of superficial cells becomes smaller. Our aim was to define an area which allows a suitable differentiation of intermediate from superficial cells in at least 95% of cases. To define a precise threshold, vaginal smears from 10 bitches in late proestrus or estrus were selected. On each specimen, the nuclei of 20 cells with at least two cornification lines and a definable and demarcated nucleus = defined as superficial cells (see Figure 2) were measured and analyzed. This resulted in 200 nuclei in total. The measurements revealed a mean value of 57.7 μm^2^ (±13.8 μm^2^) for the area of the measured nuclei. The results of the nucleus area measurements are shown in Figure 4. The majority (95 %) of nuclei of cornified vaginal cells is smaller than 79.5 μm^2^. Therefore, the value of 79.4 μm^2^ was selected as threshold for the maximum area of the nucleus of a superficial cell. Thus, if the area of the nucleus is 79.5 μm^2^ or larger the cell is classified as intermediate cell. If the nucleus has an area of 79.4 μm^2^ or smaller the user should follow the flowline to decision box 3.a. and the cell is excluded from being an intermediate cell.

Decision box 3.a. refers to the visibility of the nucleus. If the cell has no visible nucleus, the cell is classified as a squamous cell. If the nucleus is still identifiable, the flowline leads to decision box 3.b. In this case, the grade of degeneration of the nucleus has to be determined. If “the nucleus is definable, demarcated and there is a color difference between nucleus and cytoplasm” the cell it is classified as a superficial cell. Otherwise, if “nucleus is eroded and only just visible” the cell is classified as a squamous cell.

The independent evaluation of 100 randomly selected vaginal cells by five persons using the tutorial led to a Fleiss' Kappa of κ = 0.858.

## Discussion

Vaginal cytology in dogs and its usefulness and limitations have been controversially discussed recently (15). On the one hand, this diagnostic method has been described as a valuable clinical tool in the context of gynecological examinations (12) but on the other hand, usability of this method may be low for detection of the ideal time for mating or insemination (5, 16). Other authors state the vaginal cytology allows ovulation timing only retrospectively (13, 26). In regard to these different opinions, it seems worthwhile to critically assess this alleged proved and tested method as suggested for many well-proven procedures by Fontbonne (28).

Several factors may impede the reliability of vaginal cytology, which include individual cellular characteristics of the bitch such as a variable percentage of anuclear cells at the time of ovulation (29) or an influx of other cells like neutrophils (1) or erythrocytes (5).

In addition, different methods of taking and staining the smears, as well as no standardized evaluation methods may lead to variability in the interpretation (15, 26). Some authors recommend sampling from the vaginal vestibulum. Advantages of this procedure may include the reduced risk of contamination of the vagina or trauma as well as less defense reactions of sensible bitches if the swabs are not inserted into the more cranial parts of the vagina (19). Other authors do not recommend taking the specimen from the vaginal vestibulum because its cells do not react as quickly to an increase in the blood estrogen concentration as the vaginal mucous membrane (11) and are, therefore, not as indicative of the stage of the estrus cycle (7). Whether the use of a speculum improves the reliability of vaginal cytology and which staining method leads to the most robust evaluation results, has yet to be clarified (15). Also the staining of the cells with different stains need to be discussed. Diff Quick is a rapid, modified Wright- Giemsa stain that is easy to use in a clinical setting (16) and widely used for vaginal smears (15). Therefore, this stain was used for this project. Other stains such as Papanicolaou or Shorr (modified Papanicolaou) are able to detect eosinophilic cells by staining them orange- red (4). This simplifies the identification of superficial cells (4). These stains, however, are not widely used in practice because of the high costs and time requirements (15). If the results of our project would have been different with other staining remains open.

Furthermore, it seems that an important reason for the low reliability is the above-named ambiguity of definitions of different authors for vaginal cells. Arlt (15) has showed highly variable results of vaginal smear assessment probably caused by subjective evaluation.

For preparation of new cell definitions, scientific literature and experts' opinions were analyzed which revealed interesting insights into the perils and pitfalls of vaginal cytology. The Café Reprod E-mail list was chosen for the distribution of the survey to reach participants with a high level of experience in small animal reproduction. Indeed, 16 respondents cannot be regarded as representative. Nevertheless, according to their statements, most were quite experienced veterinarians in the field of small animal reproduction. It can be assumed that a selection bias needs to be considered, namely that people more interested in vaginal cytology were probably more likely to participate in the survey. In that regard, the results of the survey are even more surprising. The agreement of the raters regarding the definition of the vaginal cells was around κ = 0.4. This means poor agreement. A value of 0.0 indicates an agreement not better than chance, values lower than 0.40 indicate poor agreement, higher than 0.75 good agreement and 1.0 perfect agreement (30).

The participants stated that cell determination has a low and progesterone measurement has a high reliability in the context of ovulation timing. Nevertheless, still 13 out of 16 routinely perform vaginal cytology. Arlt (15) found similar results in a similar survey. When using both methods, veterinarians may rely more on the progesterone measurement than on vaginal cytology because it is easier and quicker to interpret. The effort on evaluating and interpreting vaginal smears and, therefore, the experience and routine to do so may have decreased among practitioners. This, in turn, may be a reason why the determination of exfoliated vaginal cells is considered to be unreliable. The question arose if more experienced raters may have a lower variation. Therefore, only raters who stated to examine more than 100 bitches per year were included in a subgroup evaluation. This led to a Fleiss' Kappa of κ = 0.533, meaning moderate agreement. This analysis suggests that variations between the raters may decline with growing experience.

Nevertheless, it is to mention that the raters did not evaluate the stage of estrus cycle based on a whole smear in this project. They were asked to name single vaginal cells.

It seems that some types of cells are easier to determine than other cells. Angular, cornified cells with a definable nucleus or no visible nucleus are better assignable for the raters, almost regardless of the level of experience. However, cells with an oval to polygonal shape, a vesicular and big nucleus and without or little cornification seem to be difficult to define, even for experts. A possible explanation for this result could be that the evaluation based on cornification and a disappearing or pyknotic nucleus is easier to recognize than the evaluation based on the size of a cell and nucleus since there is a scale necessary. In addition, the determination of cells as basal cell by various respondents, independent of their level of experience, seem noteworthy because of their questionable occurrence in a vaginal smear. The characterization of basal cells seems obsolete according to several authors since these cells build the lowest layer of the mucosal membrane (5, 10, 16)and usually cannot be collected with a swab without harming the vaginal epithelium. Therefore, this type of cell is not included in this project.

To improve the reliability of vaginal cytology, standard operating procedures for the interpretation of vaginal smears may be helpful (15). In 1967, Schutte published a classification of vaginal cells. He assigned the cells to groups A to D (19). Limitation of this classification include the widespread thresholds for the nuclear diameter. For example, Schutte stated that the diameter of a large intermediate cell ranges from 7 to 11 μm and that the diameter of a superficial cell is smaller than 6 μm. A definition of cells with a nuclear diameter between 6.0 μm and 6.9 μm was not given. In addition, some definitions such as “small intermediate cell have a relatively large nucleus” were not precise. Noteworthy is, however, that the classification of Schutte has been published 55 years ago and he had not the same technical possibilities for scanning and measuring vaginal cells as we have today. In this project, opinions and experiences of different authors and experts as well as measurements made with the program QuPath^®^ were combined. The tutorial aims to support evaluators analyzing and defining vaginal cells. The evaluation of the tutorial with experienced and unexperienced raters led to a high inter observer agreement. The Fleiss-Kappa κ = 0.858 can be interpreted as good agreement. A limitation of this project is that only five raters tested the tutorial so far. Since two students used the tutorial with good results, it can be postulated that the tutorial is user-friendly and supports determination of the cells also for non-experts. A debatable point is that the cells in the evaluation were not determined by raters beforehand without the tutorial. However, one can assume that a certain training effect would have biased a control examination, especially if the tutorial would have been offered before a non-tutorial evaluation. Another limitation of this project is the applicability and practicality of the tutorial in daily practice which needs to be tested in future studies. The effort of measurements of cells under the microscope has been described as time-consuming and unsuitable (22). This also applies for the process of digitalization. The measurement of the size of cells or nuclei can be difficult and time consuming. Nevertheless, the regular use of the tutorial may lead into a certain training effect, which was also observed during the evaluations during this project.

If the tutorial is useful in the context of ovulation timing needs to be further assessed in future studies. In the context of this project, the focus was set on the definition of cells. The cell patterns in relation to ovulation or in the context of specific gynecological disorders was not assessed. To limit a breed–related bias the authors strived a preferably wide diversity of breeds (*n* = 27).

To date, the scan process of a slide for this project lasted about 20 min. The file size of the data of one scanned smear was two to three Gigabytes. The quality of the scan highly depends on the quality of the smear. Especially specimens from bitches in anestrus or early proestrus often include only few, small cells compared with specimens from bitches in estrus and early diestrus. In consequence, the scanner has less areas to focus on and less sharp digital scans can be the result. Other variables, such as inconsistent staining and low color contrast, folded cells, air bubbles and particles may also lead to scans of low quality. While human evaluators can compensate these limitations to a certain extent by “reading through” them (31), an automated slide evaluation might not lead to appropriate results. Therefore, accurate collection of cells and preparation of slides, including the proper application of cell material, correct fixation of the cover slips, wiping the slides before scanning, are important requirements for a successful scanning process result (32). Thus, to date the scanning and measurement procedures presented are not usable in daily practice. In practice, scales in the ocular of microscopes may omit the need for digitization of the smears. Parameters from the tutorial can still be used. If a rough assessment of diameters and sizes leads to good agreements needs to be tested in future studies.

Advantages of digital microscopy like remote and off-site access to digitalized slides, easy handling, improved ergonomics, and quantitative measurements are evident (33). New microscopes, which allow easy digitalization and connection to computers or mobile devices, are already available and it can be expected that they will undergo a rapid further development. Therefore, it is likely that new measurement procedures or even automated cell evaluations will be possible in the near future. Partial scanning of smears with a minimum number of cells to decrease the duration of the scan process and the file size could raise the practicality. More standardized determination of vaginal smears and the emerging digitalization technologies may lead to a more reliable use of vaginal cytology. Another scenario could be an automated evaluation of vaginal smears by artificial intelligence (AI). There is a strong public interest and market forces that are driving the rapid development of such diagnostic procedures (34). Also in challenging on-site staffing situations, such as the COVID 19 pandemic, digital microscopy may be an important tool to keep histology workflows running smoothly (35). Some studies have shown that AI is even partially superior to human experts in cytology, namely in determining neoplastic vs. normal cells (36). Based on AI, standardized diagnostic procedures are possible which minimizes bias from the experience of the evaluators, laboratory equipment and other factors. Potential positive consequences may include reduced costs and earlier diagnosis (37). In digital pathology it is already stated that computerized analysis of specimen based on AI has the potential to reduce laborious tasks while minimizing interobserver variability and maximizing reproducibility (38). AI nowadays can also be used routinely for fecal screening for parasitic infections. Scanners automatically capture images from specimens and upload them into a cloud where the images are processed and analyzed for intestinal parasite eggs. The whole process is comparable or even quicker than the preparation time for conventional fecal flotation tests and led to agreeable results on comparison between the scanner system and parasitologists' examinations (39). Based on these developments, it seems realistic that computer-based analysis of vaginal smears in conjunction with reliable definitions of cell types are possible in the near future. Potentially, the usefulness of vaginal cytology may be improved and should be re-evaluated in the context of detection of gynecological disorders and ovulation timing. Further research is required to study if AI is helpful for the evaluation of vaginal smears. In addition, it needs to be tested if this will allow a more precise determination of the cycle stage. Whether the exact ovulation is predictable by an objective evaluation based on this tutorial has to be assessed in further studies.

## Conclusion

Vaginal cytology is a useful tool for cycle staging and breeding management of female dogs because of its quick results and easy application. Nevertheless, the evaluator needs to follow standardized determination procedures to obtain objective and repeatable results. In that regard revised cell definitions and a tutorial were developed. In future steps we aim to develop methods for computer-based analysis of vaginal smears.

## Data Availability Statement

The original contributions presented in the study are included in the article/supplementary material, further inquiries can be directed to the corresponding author.

## Ethics Statement

The animal study was reviewed and approved by Dr. Mechthild Wiegard; Ethics Commissioner of the Freie Universität Berlin. Written informed consent for participation was not obtained from the owners because written informed consent was waived for this study, because all vaginal smears were taken during gynecological examination in the context of ovulation timing or routine gynecological health check by trained experts.

## Author Contributions

FR and SA contributed to conception and design of the project and wrote the manuscript. FR organized the database, the survey, and the flowchart. RK had a consultative role and digitized the vaginal smears. VB performed the statistical analysis. All authors contributed to manuscript revision, read, and approved the submitted version.

## Conflict of Interest

The authors declare that the research was conducted in the absence of any commercial or financial relationships that could be construed as a potential conflict of interest.

## Publisher's Note

All claims expressed in this article are solely those of the authors and do not necessarily represent those of their affiliated organizations, or those of the publisher, the editors and the reviewers. Any product that may be evaluated in this article, or claim that may be made by its manufacturer, is not guaranteed or endorsed by the publisher.
